# Preoperative nutritional support in patients undergoing pancreatic surgery affects PREPARE score accuracy

**DOI:** 10.3389/fsurg.2023.1275432

**Published:** 2023-11-17

**Authors:** Pavel Skalicky, Katerina Knapkova, Jana Tesarikova, Michal Gregorik, Dusan Klos, Martin Lovecek

**Affiliations:** ^1^ Department of Surgery I, Faculty of Medicine and Dentistry, Palacky University Olomouc, Olomouc, Czech Republic; ^2^ Department of Surgery I, University Hospital Olomouc, Olomouc, Czech Republic

**Keywords:** prognostic risk score, nutritional support, morbidity, PREPARE, pancreatic resection

## Abstract

**Background:**

This study aimed to validate the accuracy of the Preoperative Pancreatic Resection (PREPARE) risk score in pancreatic resection patients.

**Patients and methods:**

This prospective study included 216 patients who underwent pancreatic resection between January 2015 and December 2018. All patients in our cohort with weight loss or lack of appetite received dietary advice and preoperative oral nutritional supplementation (600 kcal/day). Demographic, clinicopathological, operative, and postoperative data were collected prospectively. The PREPARE score and the predicted risk of major complications were computed for each patient. Differences in major postoperative complications were analyzed using a multivariate Cox proportional hazards regression model. The predicted and observed risks of major complications were tested using the C-statistic.

**Results:**

The study included 216 patients [117 men (54.2%)] with a median age of 65.0 (30.0–83.0) years. The majority of patients were classified as American Society of Anesthesiologists (ASA)’ Physical Status score II (*N* = 164/216; 75.9%) and as “low risk” PREPARE score (*N* = 185/216; 85.6%) before the surgery. Only 4 (1.9%) patients were malnourished, with albumin levels of less than 3.5 g/dl. The most common type of pancreatic resection was a pylorus-preserving pancreaticoduodenectomy (*N* = 122/216; 56.5%). Major morbidity and 30-day mortality rates were 11.1% and 1.9%, respectively. The type of surgical procedure (hazard ratio [HR]: 3.849; 95% confidence interval [CI]: 1.208–12.264) and ASA score (HR: 3.089; 95% CI: 1.067–8.947) were significantly associated with the incidence of major postoperative complications in multivariate analysis. The receiver operating characteristic curve was 0.657 for incremental values and 0.559 for risk categories, indicating a weak predictive model.

**Conclusion:**

The results of the present study suggest that the PREPARE risk score has low accuracy in predicting the risk of major complications in patients with consistent preoperative nutritional support. This limits the use of PREPARE risk score in future preoperative clinical routines.

## 1. Introduction

Monitoring perioperative morbidity and mortality is fundamental in evaluating surgical care quality. In addition to its health impact on patients’ short- and long-term survival, it has consequences in the form of higher care costs. While complications occur in 3%–17% of all surgical procedures (1, 2), the incidence is up to 60% higher in pancreatic resections (3–6).

Due to the recent increase in the aging population, there has been an increase in the total number and proportion of older patients undergoing pancreatic surgery in recent decades (7–10). The risk of postoperative complications and death is significantly higher in older patients owing to various factors, such as multiple chronic diseases and general frailty. One way to reduce the morbidity associated with surgery and improve the quality of life of patients with pancreatic disease is to seek an accurate method for preoperative risk estimation.

General scoring systems for risk stratification, such as the American Society of Anesthesiologists’ Physical Status (ASA) score (11) or the Physiological and Operative Severity Score for the Evaluation of Morbidity and Mortality (POSSUM) (12) cannot accurately predict morbidity and mortality after pancreatic resection (13, 14). Braga et al. (15) and Greenblatt et al. (16) published several pancreaticoduodenectomy (PD)-specific scores combining preoperative and intraoperative variables. The limitations of the above scores are the large number of variables (up to 21 predictors) and the use of intraoperative characteristics that exclude these scores to stratify patients preoperatively.

In 2014, Uzunoglu et al. presented an easily applied scoring system based on eight independent preoperative assessable variables to identify low- and high-risk pancreatic resection patients (17). This scoring system was based on data collected from four high-volume centers. For the widespread use of any predictive risk scoring system in daily practice, its validation in diverse patient groups, with differences in preoperative patient preparation, pancreatic anastomosis technique, drainage methods, and postoperative management, is extremely important. Only two studies focused on Preoperative Pancreatic Resection (PREPARE) score validation in an external set of patients have been published worldwide (18, 19). This study aimed to validate the accuracy of the PREPARE risk score in a population of 216 patients who underwent pancreatic resection in a high-volume hospital in Central Europe. Our findings will help use the PREPARE risk score carefully in future preoperative clinical routines.

## 2. Materials and methods

### 2.1. Patient selection

A prospective cohort study of 216 consecutive patients who underwent pancreatic resection at the Department of Surgery, University Hospital Olomouc, Czech Republic, was conducted between January 2015 and December 2018. Pancreatic and periampullary (ampulla of Vater, distal bile duct, duodenum), malignant and benign patologies were indication for surgery (Table 1). The types of pancreatic resections included in this study were PD in Whipple modification, pylorus-preserving pancreaticoduodenectomy (PPPD) in Traverso modification, distal pancreatectomy (DP), and total pancreaticoduodenectomy (TP). This study was approved by the Institutional Ethics Committee of University Hospital Olomouc (approval number: 159/16), and all enrolled patients provided written informed consent. All patients were examined by a nutritionist at the time of indication for surgical resection. If they met the following criteria: (1) any weight loss in the last six months or (2) lack of appetite; these patients received dietary advice and preoperative oral nutritional supplementation (600 kcal/day). The remaining patients did not undergo any specific nutritional intervention.

**Table 1:** Demographic, operative and postoperative characteristics of all patients (*N* = 216) stratified by PREPARE risk score.

Variables	Low risk (*N* = 185, 85.6%)	Intermediate risk (*N* = 29, 13.4%)	High risk (*N* = 2, 0.9%)	All patients (*N* = 216, 100.0%)
Age (years)				
<61	57 (30.8%)	9 (31.0%)	0 (0.0%)	66 (30.6%)
61–70	65 (35.1%)	13 (44.8%)	0 (0.0%)	78 (36.1%)
>70	63 (34.1%)	7 (24.1%)	2 (100.0%)	72 (33.3%)
Sex				
Male	98 (53.0%)	17 (58.6%)	2 (100.0%)	117 (54.2%)
Female	87 (47.0%)	12 (41.1%)	0 (0.0%)	99 (45.8%)
Histology				
Pancreatic adenocarcinoma	110 (59.5%)	14 (48.3%)	0 (0.0%)	124 (57.4%)
Chronic pancreatitis	19 (10.3%)	1 (3.4%)	0 (0.0%)	20 (9.3%)
Carcinoma ampulla of Vater	10 (5.4%)	5 (17.2%)	1 (50.0%)	16 (7.4%)
Neuroendocrine tumor	13 (7.0%)	0 (0.0%)	0 (0.0%)	13 (6.0%)
Cholangiocarcinoma	6 (3.2%)	5 (17.2%)	1 (50.0%)	12 (5.6%)
IPMN	6 (3.2%)	1 (3.4%)	0 (0.0%)	7 (3.2%)
Operative time (min)	289 (93–457)	300 (131–360)	349.5 (347–352)	293 (93–457)
Hospital stay (days)	11 (5–88)	12 (5–62)	10 (9–11)	11 (5–88)
Blood loss (ml)	400 (100–2,000)	500 (200–1,200)	450 (300–600)	400 (100–2,000)
Mortality				
In-hospital	4 (2.2%)	0 (0.0%)	0 (0.0%)	4 (1.9%)
30-day	4 (2.2%)	0 (0.0%)	0 (0.0%)	4 (1.9%)
90-day	4 (2.2%)	1 (3.4%)	0 (0.0%)	5 (2.3%)
POPF				
Grade B	11 (5.9%)	2 (6.9%)	0 (0.0%)	13 (6.0%)
Grade C	4 (2.2%)	1 (3.4%)	0 (0.0%)	5 (2.3%)
DGE				
Grade A	13 (7.0%)	2 (6.9%)	0 (0.0%)	15 (6.9%)
Grade B	3 (1.6%)	1 (3.4%)	0 (0.0%)	4 (1.9%)
Grade C	1 (0.5%)	0 (0.0%)	0 (0.0%)	1 (0.5%)
PPH				
Grade A	2 (1.1%)	0 (0.0%)	0 (0.0%)	2 (0.9%)
Grade B	2 (1.1%)	2 (6.9%)	0 (0.0%)	4 (1.9%)
Grade C	4 (2.2%)	1 (3.4%)	0 (0.0%)	5 (2.3%)
Clavien-Dindo				
Grade 0	96 (51.9%)	11 (37.9%)	1 (50.0%)	108 (50.0%)
Grade I	28 (15.1%)	1 (3.4%)	1 (50.0%)	30 (13.9%)
Grade II	43 (23.2%)	11 (37.9%)	0 (0.0%)	54 (25.0%)
Grade IIIa	2 (1.1%)	1 (3.4%)	0 (0.0%)	3 (1.4%)
Grade IIIb	8 (4.,3%)	3 (10.3%)	0 (0.0%)	11 (5.1%)
Grade IVa	4 (2.2%)	0 (0.0%)	0 (0.0%)	4 (1.9%)
Grade IVb	4 (2.2%)	2 (6.9%)	0 (0.0%)	6 (2.8%)

Qualitative data are expressed as *n* (%) and quantitative data as median (min-max). DGE, delayed gastric emptying; POPF, postoperative pancreatic fistula; PPH, postoperative pancreatic hemorrhage.

### 2.2. PREPARE score calculation

Based on the method described by Uzunoglu et al., we used the model for calculating the PREPARE score and estimating the predicted risk of major complications (17). The calculation was performed preoperatively for each patient enrolled in the study using physiological (albumin and hemoglobin levels, ASA score, heart rate, and systolic blood pressure) and operative variables (elective or emergent surgery, type of surgery, and pancreatic or nonpancreatic origin of disease) using preoperative physiological and blood parameters closest to the time of surgery, preferably obtained on the last preoperative day. The categories of each score component are summarized in Table 2, the range of the PREPARE predictive score values was 0–19 points. For risk assessment, patients were divided into 3 groups – low risk (*<*6 points), intermediate risk (6–9 points) and high risk (*>*9 points).

**Table 2:** Variables included in the PREPARE risk score and their representation in our cohort of patients.

Variables	Categories	Risk score points	Number of patients (*N* = 216, 100%)
Albumin level (g/dl)	<3.5	5 points	4 (1.9%)
	≥3.5	–	212 (98.1%)
Elective surgery	Yes	–	215 (99.5%)
	No	4 points	1 (0.5%)
Surgical procedure	PD/PPPD	2 points	135 (62.5%)
	DP	–	59 (27.3%)
	TP	–	22 (10.2%)
Pathology of pancreatic origin	Yes	–	173 (80.1%)
	No	2 points	43 (19.9%)
Heart rate (bpm)	50–80	–	185 (86.6%)
	<50 or >80	2 points	31 (14.4%)
Systolic blood pressure (mmHg)	110–130	–	114 (52.8%)
	<110 or >130	2 points	102 (47.2%)
Hemoglobin level (g/dl)	11.5–17	–	193 (89.4%)
	<11.5 or >17	1 point	23 (10.6%)
ASA score	I/II	–	186 (86.1%)
	III/IV	1 point	30 (13.9%)

Qualitative data are expressed as *n* (%). ASA, American society of anesthesiologists; DP, distal pancreatectomy; PD, pancreaticoduodenectomy; PPPD, pylorus-preserving pancreaticoduodenectomy; TP, total pancreatectomy.

### 2.3. Post-operative follow-up, outcomes, and complications

The prospectively maintained database contained all data, including the type of surgery, operative time, blood loss, length of hospital stay, mortality, and complications classified according to the Clavien–Dindo (CD) classification (20). CD III-V complications were graded as major complications. The International Study Group for Pancreatic Surgery (ISGPS) definitions were used to classify postoperative pancreatic fistula (POPF), delayed gastric emptying (DGE), and post-pancreatectomy hemorrhage (PPH) (21–23). In-hospital, 30-day and 90-day mortalities were defined as patient deaths during primary hospitalization, or during the first 30 and 90 days after primary surgery.

### 2.4. Statistical analysis

Categorical variables are presented as absolute numbers and percentages, and continuous variables are expressed as a median and minimum-maximum range. The data normality was checked using the Shapiro–Wilk test. Differences in postoperative complications were analyzed using a multivariate Cox proportional hazards regression model. Hazard ratios (HRs) were presented with 95% confidence intervals (CIs), and a two-sided *p*-value of 0.05 was considered significant. The predicted and observed risks of major complications were tested using the C-statistic. IBM SPSS Statistics version 22 was used for statistical analysis.

## 3. Results

A total of 216 patients were included in this study, including 117 men (54.2%) and 99 women (45.8%). The median age of the operated patients was 65.0 (30.0–83.0) years. In the preoperative evaluation of the ASA score, the majority of patients were evaluated as ASA II (*N* = 164/216; 75.9%), and a few patients were ASA I (*N* = 22/216; 10.2%) and ASA III (*N* = 30/216; 13.9%). A total of 140 (64.8%) patients met the criteria and preoperative nutritional preparation was indicated. Most patients (*N* = 185/216; 85.6%) were classified as low risk according to the PREPARE score. A summary of the demographic and clinicopathological characteristics of all the risk groups is presented in Table 2.

An overwhelming majority of patients (*N* = 173/216; 80.1%) underwent surgery because of a disease of pancreatic origin, and only one patient met the criteria for emergency surgery. The most common type of pancreatic resection was PPPD (*N* = 122/216; 56.5%), followed by DP (*N* = 59/216, 27.3%), TP (*N* = 22/216, 10.2%), and PD (*N* = 13/216, 6.0%). Only 4 (1.9%) patients were categorized as malnourished, with albumin levels of less than 3.5 g/dl (Table 1).

Among the specific pancreatic complications, POPF grades B and C occurred in 13 (6.0%) and five (2.3%) patients, respectively; PPH grades A, B, and C occurred in two (0.9%), four (1.9%), and five (2.3%) patients, respectively. The incidence of postoperative morbidity was as follows: CD 0, 108 (50.0%); CD I, 30 (13.9%); CD II, 54 (25.0%); CD IIIa, 3 (1.4%); CD IIIb, 11 (5.1%); CD IVa, 4 (1.9%); and CD IVb, 6 (2.8%). The 30-day and 90-day mortality rates in the entire cohort were 4 (1.9%) and 5 (2.3%) patients, respectively (Table 1).

**Figure 1:**
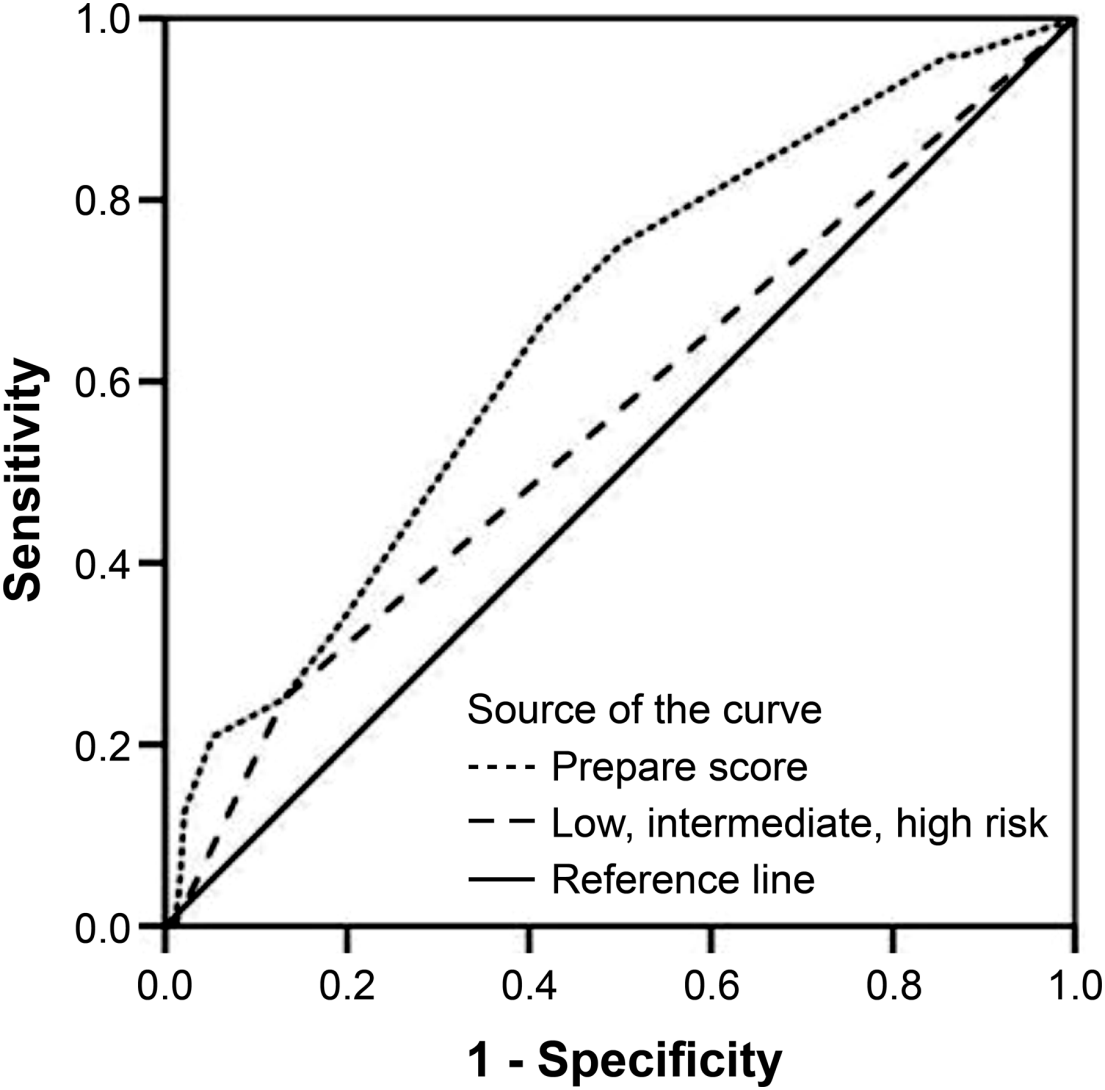
Prediction of risk for major complications, receiver operating characteristic curve in all patients (*N* = 216); score as incremental values: C-statistic index = 0.657; 95% CI: 0.544–0.771, *P* = 0.012; score as three risk categories: C-statistic index = 0.559; 95% CI: 0.430–0.687, *P* = 0.350). CI, confidence interval.

In the multivariate regression model, the type of surgical procedure (HR: 3.849; 95% CI: 1.208–12.264) and the ASA score (HR: 3.089; 95% CI: 1.067–8.947) were independent determinants of major postoperative complications. None of the other PREPARE score components showed any statistical relevance on major postoperative complications occurrence (Table 3). The observed-to-predicted (O:P) ratio in terms of major morbidity stratified according to the PREPARE score was between 0.504 (PREPARE score <3) and 0.700 (PREPARE score level 3–4). In effect, the PREPARE score overpredicted major morbidities (Table 4). The predictive ability of the PREPARE score provided an area under the curve (AUC) of 0.657 (95% CI: 0.544–0.771) for scores as incremental values and an AUC of 0.559 (95% CI: 0.430–0.687) for scores as the three risk categories (Figure 1).

**Table 3:** Multivariate analysis of PREPARE score components as risk factors for major morbidity.

Variables	Category	Hazard ratio	95% CI	*P*
Albumin level (g/dl)	<3.5 vs. ≥3.5	1.454	0.101–21.017	0,784
Elective surgery	No/Yes	NA	NA	
Type of surgical procedure	PD/PPPD vs. DP/TP	3.849	1.208–12.264	**0**.**023**
Pathology of pancreatic origin	No/Yes	2.200	0.817–5.928	0.119
Heart rate (bpm)	<50 or >80 vs. 50–80	2.875	0.962–8.596	0.059
Systolic blood pressure (mmHg)	<110 or >130 vs. 110–130	0.930	0.367–2.358	0.879
Hemoglobin level (g/dl)	<11.5 or >17 vs. 11.5–17	0.194	0.023–1.622	0.130
ASA score	III/IV vs. I/II	3.089	1.067–8.947	**0**.**038**

Differences in major morbidity were analyzed using a multivariable Cox-regression model. ASA, American society of anesthesiologists; CI, confidence interval; DP, distal pancreatectomy; PD, pancreaticoduodenectomy; PPPD, pylorus-preserving pancreaticoduodenectomy; TP, total pancreatectomy.

Bold values are statistical significance.

**Table 4:** Stratification of morbidity according to PREPARE score .

Prepare score value	Number of patients	Major morbidity (predicted)	Major morbidity (observed)	O:P ratio
≤2	102 (47.2%)	11.7%	5.9%	0.504
3–4	69 (31.9%)	20.7%	14.5%	0.700
5–6	30 (13.9%)	30.2%	16.7%	0.553
≥7	15 (6.9%)	49.7%	33.3%	0.670

Qualitative data are expressed as *n* (%). O:P ratio, observed: predicted ratio.

## 4. Discussion

Surgeons and other specialties in the perioperative team use risk scores to predict the complications of pancreatic resections, which may help them to make important decisions to optimize perioperative management. Scoring systems, including the PREPARE score, built on purely preoperative variables allow for truly informed consent of patients who may be at higher risk, or risk reduction through targeted preoperative preparation. In order to achieve this goal, the scoring system must have a high accuracy. Given the very limited number of external patients on whom the PREPARE risk score has been validated to date, we performed a validation using the largest cohort to date of 216 patients operated on at a single center, with standardized preoperative nutritional screening, nutritional support, and surgical technique.

Nutritional status is a major contributor to overall survival and quality of life in cancer patients, with malnutrition being the cause of death in a significant proportion, rather than cancer itself (24, 25). The albumin level was set as a heavily weighted component of the PREPARE score. The cutoff level at 3.5 g/dl is similar to the Glasgow prognostic score (26); some other scores have lower cutoff levels at 2.5 and 3.0 g/dl (27). A high albumin level cannot guarantee optimal nutritional conditions for extensive surgery. The ISGPS nutritional recommendations do not use albumin levels for nutritional status assessment and favor weight loss and body mass index for patient evaluation (28). Using preoperative computed tomography (CT) to detect sarcopenia in malnourished and frail patients seems promising. This has the advantage of obtaining data from CT images, which is a common part of preoperative staging, and thus does not require further examination of the patient (29).

Malnutrition treatment may play an important role in reducing postoperative morbidity (28, 30, 31). A recently published prospective randomized trial on preparations using immunonutrition before PD did not show an effect compared to standard therapy (32). In contrast, nutritional preoperative preparation in conjunction with preoperative exercise therapy has been shown to positively affect albumin levels and the incidence of postoperative complications (33, 34). A common form of nutritional preparation is oral sipping at a dose of 600 kcal/day, which our group of patients also used. The defined inclusion criterion for nutritional supplementation in our cohort was any preoperative weight loss or loss of appetite, and approximately two-thirds of patients met these criteria. This consistent preoperative nutritional support factor can explain the small number of patients with low albumin levels in our set of patients and the non-significant correlation between albumin levels and major complications.

Many published studies have shown that the incidence of POPF, PPH, and major complications in patients with PD and PPPD is significantly higher than that in patients with DP, including those operated on using minimally invasive techniques (35–40). The type of surgical procedure was a component of the PREPARE score and was confirmed as a statistically significant risk factor in our multivariate analysis. The major complication rate in the PD/PPPD group was 14.8%, compared to 4.9% in the DP/TP group.

Although we did not confirm that the pathology of pancreatic origin was a risk factor in our multivariate analysis (*P *= 0.119), it plays a significant role in POPF and PPH occurrence. While patients with pancreatic ductal adenocarcinoma or chronic pancreatitis may have inflammatory and fibrotic changes due to pancreatic duct obstruction, patients with distal cholangiocarcinoma or duodenal carcinoma do not have these changes, and a soft pancreas in “non-pancreatic” pathology is the cause of more frequent anastomotic complications. This assumption has been confirmed by several published studies comparing short-term PD/PPPD results between patients with pancreatic and nonpancreatic pathology (41–46).

The ASA score alone has low accuracy in predicting postoperative complications (47). However, it is currently used as a part of complex predictive models in combination with other variables (15). Although the data presented by Uzunoglu et al. (17) did not show a statistically significant association between the ASA score and major complications (*P *= 0.135), the ASA score was determined to be a component of the PREPARE score. Our results supported that the ASA score was a statistically significant risk factor (HR = 3.089).

Our data showed low accuracy of the PREPARE score in major complication risk prediction. Various arguments exist regarding this finding. We identified significant differences when comparing our set of patients to those in the original study published by Uzunoglu et al. (17) in terms of morbidity and mortality. The major morbidity rates were 11.1% vs. 31.3% and 30-day mortality rates were 1.9% vs. 5.6%. These differences may be related to the variability of the cohorts in relation to the indication and the representation of individual types of resections. Given the universal nature of the PREPARE risk score, which should be applicable to all types of pancreatic resections, its accuracy should not be affected with this fact. At the same time, it is evident that our patient cohort was less “risky” as related to PREPARE score, as the majority (85.6%) of patients are classified as “low risk.” The albumin level was set as a heavily weighted component of the PREPARE score (5 points), and almost all “malnourished” patients are categorized as “intermediate” or “high.” The proportion of patients with albumin levels less than 3.5 g/dl was 1.9% in our cohort and 17.0% in the cohort published by Uzunoglu et al. The original data published by Uzunoglu et al. (17) presented the types of surgical procedures in PD, PPPD, DP, TP, and other resections. Several technical aspects, such as vascular or multi-visceral resection, can significantly influence morbidity within these subgroups and preclude patient cohort comparison. Vascular and multi-visceral resection rates were 3.2% and 1.4%, respectively. It seems advisable to specify these attributes when reporting pancreatic resections in the future using a classification recently published by Mihaljevic et al. (48). The lack of a more detailed specification of the resection procedure affecting the calculation of the PREPARE score is another limitation to its accuracy.

The results of the original patient validation cohort published by Uzunoglu et al. reported the accuracy of the PREPARE score in predicting major complications, with an AUC of 0.711 for incremental values and 0.709 for risk categories, which was not a strong prediction. A weakness of the above-mentioned validation may have been due to the use of data from patients operated on in the same hospital but during a different period. To date, only two studies validating the accuracy of the PREPARE score have been published worldwide. One of them, published by Celik et al. (18), was a retrospective analysis of a cohort of 122 patients and showed low prediction accuracy (AUC 0.541), whereas a later published prospective study by Rodriguez-Lopez et al. (19) included 50 patients and showed good accuracy in predicting major morbidity (AUC 0.736). Our population of patients presented worse prediction results compared to those of Uzunoglu et al., with an AUC of 0.657 for incremental values and 0.559 for risk categories, which is a weak predictive model.

To the best of our knowledge, this is only the second prospective study to focus on PREPARE score validation in an external set of patients worldwide, with the largest cohort of patients and the only study conducted in a cohort of patients with consistent preoperative nutritional support. Our study has several limitations. This was a single-center observational study with a limited number of patients. Regarding the criteria for calculating the PREPARE score, including the physiological and laboratory data collected as close as possible to those of the surgical procedure, we did not have data on albumin levels before initiating nutritional intervention in patients. Thus, we could not conduct a more detailed analysis of its impact on this nutritional parameter.

## 5. Conclusion

In summary, the present study suggests that the PREPARE risk score has low accuracy in predicting major complication risks in patients with consistent preoperative nutritional support. This limits the use of PREPARE risk scores in future preoperative clinical routines. More external score validations are needed, and given the increasing representation of patients with systematic nutritional preparation, the PREPARE score calculation parameters may need to be adjusted.

## Data Availability

The original contributions presented in the study are included in the article/Supplementary Material, further inquiries can be directed to the corresponding author.
